# Relationship between DNA replication and the nuclear matrix

**DOI:** 10.1111/gtc.12010

**Published:** 2012-11-08

**Authors:** Rosemary H C Wilson, Dawn Coverley

**Affiliations:** Department of Biology, University of YorkHeslington, York, YO10 5DD, UK

## Abstract

There is an extensive list of primary published work related to the nuclear matrix (NM). Here we review the aspects that are required to understand its relationship with DNA replication, while highlighting some of the difficulties in studying such a structure, and possible differences that arise from the choice of model system. We consider NM attachment regions of DNA and discuss their characteristics and potential function before reviewing data that deal specifically with functional interaction with DNA replication factors. Data have long existed indicating that newly synthesized DNA is associated with a nuclease-resistant NM, allowing the conclusion that the elongation step of DNA synthesis is immobilized within the nucleus. We review in more detail the emerging data that suggest that prereplication complex proteins and origins of replication are transiently recruited to the NM during late G1 and early S-phase. Collectively, these data suggest that the initiation step of the DNA replication process is also immobilized by attachment to the NM. We outline models that discuss the possible spatial relationships and highlight the emerging evidence that suggests there may be important differences between cell types.

## Introduction

### The nuclear matrix

Descriptions of an insoluble proteinaceous nuclear substructure, in some ways analogous to the cytoskeleton, have existed for at least 40 years. However, the difficulties associated with studying this nuclear fraction mean that there are still many unanswered questions about structure and function; and even some residual controversy about its very existence. However, there is now a large and growing body of evidence in favor of such a nuclear substructure. Here we review the published work on its relationship with DNA replication.

The nuclear substructure has been termed the nuclear matrix (NM), the nuclear scaffold or the nuclear skeleton (or nucleoskeleton) depending on the technique used to reveal it. These are, respectively, extraction with high salt (2.0 m NaCl) (Berezney & Coffey 1974), lithium 3,5-diiodosalicylate (LIS) (Mirkovitch *et al*. 1984) or after encapsulation in agarose under physiologically relevant salt concentrations and electrophoresis (Jackson & Cook 1988). A protein is termed part of the nuclear substructure if it resists extraction. However, there are also many variations of these techniques (reviewed in Martelli *et al*. 2002), and this means that interpretation is not always straightforward. Here we use the term NM as an overarching term and aim to consolidate the reported observations to outline some general phenomena. Depending on extraction technique, the residual protein fraction varies slightly in composition but the core components revealed by several large-scale screens are similar (Albrethsen *et al*. 2009; Varma & Mishra 2011; and references therein). These include lamins, matrins, hnRNPs, other ‘structural’ proteins, and various proteins involved in DNA metabolism, many of which are listed and categorized in a database of NM Proteins, NMPdb (Mika & Rost 2005).

Very early work described a proportion of nuclear proteins as unextractable with high salt (reviewed in Martelli *et al*. 2002) however, the idea of a NM really began with electron micrographs (EM) showing a network of fibers that remain within the nucleus after extraction (Berezney & Coffey 1974). The controversy associated with NM research appears to be attributable to two key reasons. First, it has proved difficult to show such a framework as that seen by EM using immunofluorescence methods in unextracted cells. Instead immunofluorescence against candidate NM proteins usually reveals a pattern of punctate spots (reviewed in Martelli *et al*. 2002). One study used electron spectroscopic imaging (ESI) to view unextracted nuclei by electron microscopy with the aim of identifying areas as protein and/or nucleic acid rich. Using paraformaldehyde fixed sections, this showed inter chromosomal areas to be composed of protein rich but nucleic acid poor structures, consistent with the description of a NM (Hendzel *et al*. 1999). Also worth consideration when thinking about the NM is the idea that there may exist multiple local NMs (Martelli *et al*. 2002) that are dynamic and capable of altering characteristics and composition based on the nuclear processes occurring at that point in time and space (Nelson *et al*. 1986). Therefore, we might not expect to see filamentous structures composed of a small number of specific proteins, but instead transient associations between functional proteins and a ‘core’. The use of nonphysiological conditions, in particular high salt extraction, has been criticized as potentially causing aggregation of such protein assemblies (Pederson 1998). To address this, the LIS and physiologically relevant buffer techniques were developed, which give very similar results.

The second major reason that appears to have added complexity and contributed to the controversy in the NM field is the widespread use of systems that now appear to lack or possess a different kind of NM, such as *Xenopus* eggs, cancer cell lines, and progenitor cells. Recent research suggests that the NM changes as cells differentiate or become transformed (reviewed in Zink *et al*. 2004; Munkley *et al*. 2011, and discussed in more detail below).

We will not review evidence for the NM as this has been extensively evaluated elsewhere (Pederson 1998; Hancock 2000; Nickerson 2001; Martelli *et al*. 2002). Instead we consider its functional significance. The NM has been proposed as an anchor for DNA structure, the site of transcription (Jackson & Cook 1985), DNA repair (Qiao *et al*. 2001), splicing (Zeitlin *et al*. 1987), chromatin remodeling (Reyes *et al*. 1997), and DNA replication (Jackson & Cook 1986b). Here, we aim to describe how a NM could support DNA replication and discuss proposed mechanisms and models.

### Structural organization of DNA

In order to fit approximately 2 m of DNA within a mammalian nucleus of the order of 10 μm in diameter, it is clear that there must be multiple levels of organization. The extremes of packing of DNA, from wrapping the double helix around histone octamers to form nucleosomes, to compartmentalization into chromosome territories (reviewed in Cremer *et al*. 2006) are now as accepted as the helix itself. However, intermediate levels of organization incur more debate (reviewed in Bian & Belmont 2012).

One model is that nucleosomes are organized into a 30 nm fiber. However, existence of this structure *in vivo* is still hotly contested as the evidence is largely from *in vitro* work (Bian & Belmont 2012). An alternative model is that of fractal globules (reviewed in Fudenberg & Mirny 2012), in which short regions of DNA are proposed to ‘crumple’ (condense) together to form a series of globules (or domains). These then further crumple together to form larger globules, repeating until they achieve the size of chromosome territories. Some key points of this model are that DNA is not knotted as it would be in an equilibrium globule model (random compaction of DNA), and that it includes all the required levels of compaction. In this model, spatially segregated domains would suggest flexible access to each region of DNA. There are supportive data for the idea of domains within domains, from Fluorescence *In Situ* Hybridisation (FISH) and Chromosome Conformation Capture techniques (Lieberman-Aiden *et al*. 2009). Consistent with both of the above, chromatin loops are thought to periodically attach to the NM. It is likely that the true state of chromatin is a combination of these proposed higher order structures and is likely to be highly dynamic. Here we focus primarily on the organization inherent in attachment to the NM.

## Attachment of DNA to the nuclear matrix

**Figure 1:**
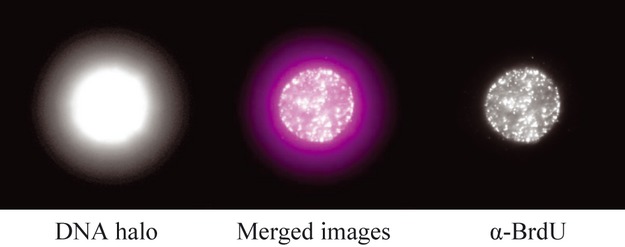
Newly synthesized DNA (A) Left: example of Maximum Fluorescence Halo Radius image from NIH3T3 cell showing DNA loops stained with DAPI emanating out from the nuclear matrix (NM) (MFHR method described in Buongiorno-Nardelli *et al*. 1982; Guillou *et al*. 2010). Right: newly synthesized DNA is observed at the NM but not visible in loop DNA. Cells were pulsed for 30 min with BrdU and visualized with α-BrdU. Centre: merged image showing BrdU (newly synthesized DNA) in white and DNA in magenta.

**Figure 2:**
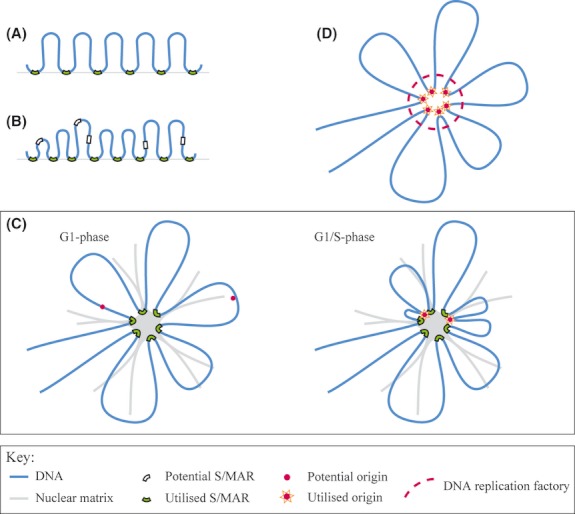
Possible relationships between DNA replication and the nuclear matrix (NM). (A) DNA is thought to be periodically attached to the NM at S/MARs forming intervening chromatin loops. (B) Refined model illustrating variable loop sizes and complex S/MAR usage including utilized S/MARs and function-related alternative potential S/MARs. (C) Our preferred model showing attachment to the NM via nonorigin S/MARs, and recruitment of DNA replication origins at G1/S-phase with possible impact on loop size. (D) Alternative representation of chromatin loops showing replication origin clustering within a DNA replication factory, with no representation of NM attachments.

Attachment to a proteinaceous structure was first observed by electron microscopy in the 1970s (Paulson & Laemmli 1977). Various methods have been used to study the DNA : NM attachment points (Mirkovitch *et al*. 1984) including imaging by FISH and Maximum Fluorescence Halo Radius (MFHR) (Fig. 1), and biochemical (digestion of loop DNA with restriction enzymes, DNase I or topoisomerase II). Early studies showing attachment of DNA to the NM led to the idea of periodic attachments and the concept of intervening chromatin loops (Benyajati & Worcel 1976; Marsden & Laemmli 1979) (Fig. 2A). Subsequent work has led to a modification to include loops of different sizes (Fig. 2B) and likely different functions (described in more detail below).

Attachments are termed MARs (matrix attached region), which are resistant to extraction with high salt, scaffold attached region (SARs), which are resistant to extraction with LIS, or skeleton-attached sequences, which are resistant to physiological buffers after encapsulation in agarose, all coupled with enzymatic digestion of DNA. The attachment points revealed by different extraction methods have significant overlap but some are method specific. For example, only approximately half of sequences revealed as MARs on chromosomes 14–18 were also identified as SARs (Linnemann *et al*. 2009). It is likely that the total attachment points in a cell comprise a combination of those revealed by these techniques. We first discuss general features of attachment regions, for which we use the overarching term S/MAR, before highlighting some differences between MARs, SARs and skeleton-attached sequences.

The length of DNA that is associated with the NM at each attachment point is variously reported as between 100 and 1000 bp with individual loops ranging from 4 to 200 kbp (Vogelstein *et al*. 1980; Buongiorno-Nardelli *et al*. 1982; Singh *et al*. 1997; Bode *et al*. 2003). From our data, we calculate an average loop size of 70 kbp in noncancer differentiated mammalian cells (Wilson, R.H.C., and Coverley, D., our unpublished observation), which ties in closely with several of the previous estimates in the 60–86 kbp range (Vogelstein *et al*. 1980; Jackson *et al*. 1990). With a human haploid genome of ∼3 billion bp, this would suggest that ∼86 000 attachment points exist at any one time per diploid, G1 cell. As different S/MARs exist in different cell types, and most likely in different phases of the cell cycle and transcriptional programmes, the total number of potential S/MARs is likely to be much more than this (Fig. 2B). Boulikas (1995) predicted approximately 100 000 potential S/MARs based on (i) a loop size of 60 kbp, (ii) an estimated genome size of 3.6 billion bp, and (iii) the fact that some S/MARs are facultative (Boulikas 1995). As we will discuss, predictive tools for S/MARs have their limitations, but it is interesting to note that all tools appear to overpredict S/MARs (Platts *et al*. 2006). While we cannot currently differentiate between false positives and facultative S/MARs, a surplus of potential S/MAR regions would allow for flexibility in usage and imply some form of selection. Consistent with these ideas, it has been shown that a sequence that has the potential to be a functional S/MAR is not always recruited to the NM (Heng *et al*. 2004).

### Features of S/MARs

Many of the S/MARs that have been identified arise from studies at a particular gene or locus, and there have been few chromosome or genome wide investigations. The collation of S/MARs identified by individual groups into the S/MAR database (Liebich *et al*. 2002a) has allowed comparison of potential S/MAR motifs (Liebich *et al*. 2002b). However, other than an over-representation of As and Ts, the authors found little sequence similarity. Instead, structural motifs are thought to play a greater role in determining the potential for NM attachment. One predictive tool, ‘MAR finder’ uses combinations of the following structural motifs to predict S/MARs; origins of replication, TG-rich sequences (commonly in 3′ UTRs), curved DNA, kinked DNA, topoisomerase II sites, and AT-rich sequences (Singh *et al*. 1997). Other suggested characteristics include transcription factor binding sites and other regulatory sequences linked with promoter function (Singh *et al*. 1997). However, some reports are contradictory and motif enrichment may depend on the method used to remove loop DNA. Moreover, the predictive power of tools that use a combination of structural motifs and AT-rich DNA is little better than prediction based on AT-content alone (Evans *et al*. 2007). One of the reasons for the lack of a good predictive model is that only a few S/MARs have been identified by different methods, which appear to identify different populations. So while a particular motif may appear common to one set of identified S/MARs, it may not be hugely predictive of S/MARs in general. It may also be that many motifs can increase the probability of a region forming NM attachment with each increasing the probability only slightly. Therefore, the identification of more S/MARs and a better understanding of their function will be necessary in order to develop better predictive tools.

### Functions of S/MARs

In spite of difficulties identifying and predicting S/MARs, several different functions have been suggested for these NM attachments. Constitutive attachments, which do not vary by cell type, are suggested to have a structural role in anchoring the DNA and maintaining nuclear architecture. Consistent with this organizational function, territories have been shown to remain after extraction to reveal the NM but be lost when the NM is disrupted with RNase (Ma *et al*. 1999). In contrast, other S/MARs appear to be transient and facultative in their attachment to the NM. Some vary by cell type and are thought to be involved in maintaining the transcriptional programe, and others vary with external signals and the subsequent change in transcriptional programe. These include S/MARs associated with transcription units, enhancers, and transcription factor binding sites. Furthermore S/MARs vary by cell cycle stage and include regions at potential origins of replication. We describe below, evidence that both transcription and replication occur at the NM. The facultative S/MARs that are implicated in these processes are likely to be involved in the recruitment of specific regions to the NM. Consistent with the idea of constitutive and facultative S/MARs, some proteins which bind S/MARs (MARBPs) are core components of the NM, such as matrins and lamins, topoisomerase II and high-mobility group proteins, while others are cell type or signal specific, such as Scaffold Attachment Factors A (SAF-A) and B (SAF-B) and SATB1 (reviewed in Wang *et al*. 2010).

Attachment regions could be grouped by characteristics, namely motif composition, extraction method or function. For example, some investigators have advocated a classification of S/MARs into the following four groups based on: regulatory elements including origins and enhancers (class I), somatic cell boundary elements (class II), haploid genome boundary elements observed in sperm (class III), and those with intermediate affinity with the NM (class IV) (Kramer & Krawetz 1996). Although this scheme does not appear to be in widespread use, it effectively classifies S/MARs by motif or structure and by context dependent factor binding.

It is possible that classification by motif or extraction method reveals the same groups. For example, matrix attachment regions (MARs) may contain specific motifs and be related to a specific function. Alternatively, one feature may be more important and, using this example, MARs may encompass regions with different function. We now describe reports from two groups that investigated whether the attachments revealed by different methods specify function.

One study used chromosome paints to compare MARs, SARs, and skeleton-attached sequences genome-wide in human lymphoblasts (Craig *et al*. 1997). Cells were extracted with either 25 mm LIS, 2.0 m NaCl or physiological buffers after agarose encapsulation, and loop fragments released using restriction enzyme digestion. Loop and attached DNA fragments were separated and fluorescently labeled and then used in combination as FISH chromosome paints. It is important to note that the resolution for this study is in the megabase range and that metaphase chromosomes were used. Nevertheless, the study found MARs to be slightly enriched in gene poor regions and relatively absent from transcriptional promoters, and SARs were also enriched in gene poor regions, whereas skeleton-attached sequences were associated with gene rich regions and CpG islands. The authors found little change in SARs between mitotic and interphase chromatin but considerable difference when looking at MARs or skeleton-attached sequences. Overall, the data suggest that SARs are constitutive and structural. In contrast, MARs appear to vary by cell cycle phase but not transcriptional status, whereas skeleton-attached sequences varied by cell cycle phase and in addition appear to be linked to transcription.

In other studies, MARs and SARs of chromosomes 14–18 of HeLa and primary aortic adventitial fibroblasts (AoAF) were compared using a microarray approach with much finer resolution than imaging approaches (Linnemann & Krawetz 2009b; Linnemann *et al*. 2009). Regions enriched in the attached DNA fraction relative to loop fraction were designated as MARs or SARs depending on the method of extraction. For both types of cell, MARs were generally found in intergenic and gene poor regions and were strongly associated with silenced genes, comparing well with data from Craig *et al*. In comparison, SARs density did not depend on gene density but tended to overlap genes and be associated with expressed genes. Linnemann *et al*. propose MARs to be structural with a subset of intragenic MARs providing silencing attachments, and SARs to be facultative and related to function, especially transcription. This suggests that control of gene expression is achieved through multiple mechanisms which include MAR attachment and histone modifications and other epigenetic marks (Linnemann & Krawetz 2009b).

The lack of more genome wide studies makes definitive statements about subgroups of S/MARs and their function difficult. However, general conclusions from these studies suggest MARs to include structural and silencing attachment points, SARs to perhaps be important for cell type determination and maintenance, and SARs and/or skeleton-attached sequences to be important for gene expression.

## DNA replication and the nuclear matrix

Various lines of evidence suggest that DNA replication occurs in association with the NM. Origins of replication are recruited to the NM, but as yet there is little indication which subgroup they are likely to fall into. Both newly synthesized DNA and termination structures exist within NM fractions, DNA replication origins appear to be facultative S/MARs, and a number of key proteins involved in replication are themselves associated with the NM. We will discuss evidence for elongation, termination, and initiation of DNA replication.

### Elongation of DNA replication

Biochemical fractionation of nuclei to localize nascent DNA, or visualization after labeling with short pulses of nucleotide analogues, has shown newly synthesized DNA and replication intermediates to be located at the NM and crucially not in the loops (Fig. 1). Furthermore, pulse-chase experiments show that when newly synthesized DNA is observed at later timepoints, it has migrated from the NM fraction into loop regions (Pardoll *et al*. 1980; Vogelstein *et al*. 1980; Jackson & Cook 1986b; Nakayasu & Berezney 1989; Gerdes *et al*. 1994). This collection of studies indicates that the DNA synthesis step and presumably therefore also replication forks are located at the NM. Consistent with this, S-phase cells possess both proliferating cell nuclear antigen (PCNA) and DNA polymerase α in the nuclear skeleton fraction (Hozak *et al*. 1993, 1994). Moreover, this population of polymerase α showed *in vitro* activity that was comparable to that *in vivo*, and nascent DNA remained associated with the skeleton (Jackson & Cook 1986a,b). Similar conclusions were drawn from analysis of NM preparations (Nakayasu & Berezney 1989).

### Termination of DNA replication

Relatively little is known about the termination of DNA replication; however, there are data that suggest an association with the NM. First, topoisomerase II is located at the NM (Berrios *et al*. 1985) and appears to be required for resolving replication intermediates. More direct evidence comes from analysis of NM attached DNA, by 2D agarose gel electrophoresis, which revealed termination structures in addition to replication intermediates. Patterns were consistent with both termination at specific points and as a consequence of the convergence of forks (Little *et al*. 1993).

### Initiation of DNA replication

The most direct evidence that initiation of DNA replication is located at the NM makes use of synchronised late G1-phase cells treated with DNase I to remove chromatin loops (Radichev *et al*. 2005). When incubated in soluble extract from S-phase cells (which contains regulatory protein kinases that induce initiation) more DNA synthesis occurred on the residual NM-associated chromatin than in control incubations that only support elongation. This indicates that NM-attached chromatin from late G1 cells can undergo initiation. Because this was found to be both located within characteristic foci and dependent on protein kinase activity, these data support the idea that the initiation step of DNA replication occurs in chromatin that is protected by association with a nuclease-resistant structure.

Interestingly, under the same conditions, chromatin-depleted early G1 nuclei did not undergo kinase dependent initiation. This may be due to incomplete assembly of all required factors of the preinitiation complex (pre-IC) or because origins are not located at the NM during early G1. Data described below do indeed suggest that origins are recruited to the NM only during late G1. However, we cannot assume that all origins are recruited to the NM together, as this may occur in conjunction with their activation.

### Origins of replication as S/MARs

In human cells, between 30 000 and 50 000 replication origins are thought to be activated per cell cycle in a spatially and temporally ordered fashion. *Saccharomyces cerevisiae* origins of replication are defined by DNA sequence, the autonomous replication sequence (ARS). However, similar short sequences do not specify origins in higher eukaryotes. Instead, structural information and epigenetic marks that can be stably inherited by daughter cells play a role in specification. These include promoter status, CpG methylation, nucleosome positioning, DNase I sensitive sites, DNA topology, and chromatin loop architecture (reviewed in Mechali 2010). For example, there is a higher probability of initiation occurring just before or after transcription start sites, with and without CpG-rich regions (Cayrou *et al*. 2011). Even in *S. cerevisiae* the ARS, though required, is not sufficient to specify origin use as contextual features are known to play a role (Nieduszynski *et al*. 2006). The apparently weak dependence on primary sequence has made it difficult to predict higher eukaryote origins, although S/MARs have been identified in the vicinity of origins of replication for a handful of exemplar genes (reviewed in Cayrou *et al*. 2010).

Early experiments suggested that mammalian cell origins were permanently associated with the NM (Lagarkova *et al*. 1998; and references therein). Furthermore, the similarity in size between replication units and chromatin loops reported in the 1980s (Buongiorno-Nardelli *et al*. 1982) suggested that they might be one and the same, with all attachment points being origins. In apparent support of this conclusion, regions of DNA labeled in early S-phase by incorporation of nucleotide analogues are observed at the NM fraction.

More recent evidence, however, suggests that recruitment of origins of replication to the NM occurs only transiently in late G1 and S-phase. Attachment to the NM and nucleoskeleton has been investigated for the well-studied origin of replication, *oriB*, at the *dihydrofolate reductase* (DFHR) locus in Chinese Hamster Ovary (CHO) cells (Ortega & DePamphilis 1998; Djeliova *et al*. 2001a,b). In one study, DNA from CHO cells was separated into loop and NM attached fractions (Djeliova *et al*. 2001a) and then probed with the *oriB* sequence or with nascent DNA from early S-phase (the collective origin fraction). No enrichment of origins, either *oriB* or the collective origin fraction, was observed in the NM attached DNA from asynchronous cells (Djeliova *et al*. 2001a). However, *oriB* was enriched in the NM attached DNA (Djeliova *et al*. 2001b) and nucleoskeleton attached DNA (Ortega & DePamphilis 1998) from late G1 phase cells (but not early G1), and lost from this fraction as cells progressed through S-phase (Djeliova *et al*. 2001b). In contrast to this early replicating origin, the late replicating origin from the *β-globin* gene was investigated in HeLa cells, where the NM association was maintained through S-phase (Djeliova *et al*. 2001b). Origins from other exemplar genes have also been described to temporally associate with the NM (reviewed in Ottaviani *et al*. 2008). Collectively, these studies show that origins have the potential to reside close to the NM, but that their association is not constitutive, being recruited during late G1 (Fig. 2C) and lost during S-phase.

### Prereplication complex components

Potential DNA replication origins are marked by the origin recognition complex (ORC1-6), then licensed by recruitment of the rest of the prereplication complex components (pre-RC), including cell division cycle 6 (CDC6), chromatin licensing and DNA replication factor 1 (CDT1) and the mini-chromosome maintenance complex (MCM2-7), and reviewed in more detail elsewhere (Takisawa *et al*. 2000; Bell & Dutta 2002). Additional proteins associate with the pre-RC to form the pre-IC, which recruits the DNA replication machinery itself (RC) (summarized in Boos *et al*. 2012). We are not aware of any published investigations of pre-IC components in relation to the NM, but several report findings for pre-RC and RC components.

Consistent with their role in the regulation of temporally restricted events, pre-RC proteins have tightly controlled temporal expression and degradation, as well as restricted subnuclear localization. Unfortunately, many researchers limit their analysis by failing to include a nuclease digestion step in cellular extraction protocols, allowing only generalized conclusions about association with chromatin and/or NM. Furthermore, the cell cycle context of potential NM recruitment means that the fraction detected may be small and their recruitment to the NM may in fact be cell-type specific making the overall picture difficult to interpret. An important consideration to highlight here is that pre-RCs are laid down at potential origins (origin licensing) during late telophase in the mitotic cell cycle (Dimitrova *et al*. 2002), but lost if cells become quiescent (Madine *et al*. 2000; Tatsumi *et al*. 2003; Cook *et al*. 2004) necessitating their re-synthesis and recruitment following cell cycle re-entry. Therefore, the timing of both pre-RC formation and their NM recruitment is likely to be different for the G1 after release from quiescence compared to the G1-phase in the mitotic cell cycle. However, the accumulation of increasingly sophisticated reports on NM attachment of pre-RC components is beginning to support the idea that some core components become attached to the NM, possibly in particular combinations and only around the time of initiation of DNA replication.

The most widely used method to determine NM recruitment of pre-RC proteins uses cytoskeletal buffer (10 mm Pipes pH 6.8, 100 mm NaCl, 300 mm sucrose, 1 mm MgCl_2_, 1 mm EGTA, 1 mm DTT) and fractionation by centrifugation (Ainscough *et al*. 2007). Soluble and insoluble proteins are recovered following treatment with 0.1% Triton X-100, and chromatin bound proteins are released by inclusion of 0.5 M or 2.0 m NaCl or subsequent treatment with DNase I. NM bound proteins are defined as those which are not released by any of these treatments. This method can be carried out on cell populations, for western blot analysis, and is also compatible with immunofluorescence-based imaging. A further distinction can be made between those proteins that are released from native cells but retained by those treated with a protein cross-linker such as DTSP. Chromatin bound proteins such as histones do not remain in the DNase I resistant fraction in the presence of cross-linker but NM associated proteins do, indicating direct interaction with a component of the NM (Fujita *et al*. 2002).

### Origin recognition complex

Data show stable levels of ORC subunits 2–5 throughout the mitotic cell cycle (Madine *et al*. 2000; Fujita *et al*. 2002; Ohta *et al*. 2003; Tatsumi *et al*. 2003; McNairn *et al*. 2005), and one report suggests this is also true for ORC1 (McNairn *et al*. 2005). However, the majority of reports show ORC1 to peak in G1 phase with subsequent proteolysis during S-phase and mitosis (Tatsumi *et al*. 2003). This ‘ORC cycle’ has been extensively reviewed elsewhere (DePamphilis 2003).

Several studies have looked at the relationship between ORC subunits and the NM. ORC1 has been detected in the NM fraction of asynchronous populations of HeLa, BJAB, and BC3 cells (Kreitz *et al*. 2001; Fujita *et al*. 2002; Ohta *et al*. 2003; Tatsumi *et al*. 2003; Ohsaki *et al*. 2009) and in *Drosophila melanogaster* cells (Varma & Mishra 2011). Several investigators have temporally resolved this NM association by cell cycle phase, which revealed ORC1 to be enriched in the NM fraction in G1, BJAB and BC3 cells, and G1 or from late G1 in HeLa cells (Kreitz *et al*. 2001; Fujita *et al*. 2002; Ohta *et al*. 2003; Tatsumi *et al*. 2003; Ohsaki *et al*. 2009). This suggests that when ORC1 is expressed it becomes NM bound. However, it should be borne in mind that NM attachment of ORC1 might be cell type dependent as one report shows ORC1 as sensitive to extraction with DNase I in proliferating NIH3T3 cells (Madine *et al*. 2000).

Other ORC subunits have also been detected in NM fractions, specifically ORC2-5 in asynchronous HeLa cells (Kreitz *et al*. 2001; Fujita *et al*. 2002; Ohta *et al*. 2003) and ORCs 2, 4, 5, and 6 in *Drosophila melanogaster* cells (Varma & Mishra 2011), although again this may be cell type specific as little ORC2 was NM bound in Raji cells (Mendez & Stillman 2000).

Where temporal resolution was attempted, ORC2-5 were enriched in the NM fraction in G1 (Kreitz *et al*. 2001) or from late G1, in HeLa cells (Ohta *et al*. 2003). In both cases, this was at the same time as ORC1 was expressed and NM bound. Furthermore investigation in HeLa cells showed that when ORC1 was depleted by RNAi, ORC2 was no longer enriched in the NM fraction (Ohta *et al*. 2003). Therefore, NM binding of ORC may well be dependent on ORC1.

### Cell division cycle 6

The majority of investigations into the subnuclear localization of the CDC6 protein have revealed a proportion that is attached to the NM. This has been noted in asynchronous HeLa cells, BJAB, BC3, and HEK293 cells and is somewhat depleted in the absence of ORC1 in FT210 cells (Fujita 1999; Fujita *et al*. 2002; Ohta *et al*. 2003; Ohsaki *et al*. 2009). However, little CDC6 was found to be NM bound in NIH3T3 cells (Madine *et al*. 2000) or Raji cells (Mendez & Stillman 2000). Where cell cycle phases were investigated, CDC6 was present in the NM fraction during G1 or early S-phase but not in G2/M cells (Fujita *et al*. 1999; Ohsaki *et al*. 2009). Therefore, it is clear that independent groups, using different methods and cell lines, have shown ORC and CDC6 to be at least transiently attached to the NM.

### Chromatin licensing and DNA replication factor 1

Along with CDC6, CDT1 is essential for the functional assembly of the MCM helicase complex and replication origin licensing (Cook *et al*. 2004). Few investigators have looked at NM attachment but one report described recovery of CDT1 in the NM fraction for BJAB, BC3, and HEK293 cells; this occurred specifically in G1 phase but not S or G2/M phases (Ohsaki *et al*. 2009). This suggests that CDT1 may be recruited to the NM but there is not enough information available to draw generalized conclusions.

### Mini chromosome maintenance complex

The heterohexameric MCM complex is believed to be the helicase responsible for unwinding DNA during replication (Takisawa *et al*. 2000), but its relationship with the NM is not as clear as for other pre-RC components. The majority of MCM3 and MCM2 is not NM bound in asynchronous HeLa cells (Todorov *et al*. 1995; Fujita *et al*. 1997) with similar results for MCM2, MCM3, and MCM5/7 in REF52 cells (Cook *et al*. 2002), MCM3 in Raji cells (Mendez & Stillman 2000), and MCM5 in NIH3T3 cells (Stoeber *et al*. 1998). However, Burkhart *et al*. (1995) report a small but significant fraction of MCM3 in HeLa cells, which is not released by nuclease or high salt extraction. There are also several reports identifying MCM4, MCM2, MCM3, and MCM7 in NM proteomic screens in human or *Drosophila melanogaster* cell lines (Gerner *et al*. 2002; Mitulovic *et al*. 2004; Varma & Mishra 2011). Although the majority of the MCM complex does not appear to be NM bound it is possible that the small amount of MCM protein in NM preparations reflects a weak, transient association, which is easily missed in bulk preparations from asynchronous cells. Some circumstantial support for the idea that attachment to the NM may be important for MCM function comes from ORC1 or CDC6 depletion studies, in which immobilization of MCM (on chromatin or NM) is prevented (Ohta *et al*. 2003). Similarly, immobilization of ORC2-5 complexes on chromatin is not sufficient for MCM2 loading in the absence of ORC1 (Ohta *et al*. 2003). As ORC1 appears to recruit the rest of the ORC complex to the NM, this begins to suggest that loading of MCM2 occurs in that fraction.

### Coordinators and regulators

A few proteins involved in the regulation of initiation of DNA replication and cell cycle progression have been studied in the context of the NM, including cyclin E (Munkley *et al*. 2011), Rb (Mancini *et al*. 1994), and CIZ1 (Ainscough *et al*. 2007).

Our study of cyclin E has been revealing, because it seeks to compare NM recruitment at different developmental stages (Munkley *et al*. 2011). In fact the NM attachment of cyclin E, a key regulator of initiation of DNA replication, may serve as a case study that goes some way to explain the differing reports of NM attachment of other replication proteins. We showed that a proportion of cyclin E is NM bound in differentiated, noncancer primary and established cell lines. However, in all but one of eight cancer cell lines we investigated, no cyclin E was observed in the NM fraction (Munkley *et al*. 2011). A similar distinction was seen between differentiated cells and undifferentiated cells in mouse, human, and *Xenopus* model systems, so that in all cases cyclin E was easily extracted from progenitor cells but resistant to extraction in differentiated derivatives. This suggests that cyclin E is recruited to the NM as cells differentiate, and that cancer cells either originate from cells that have not recruited cyclin E, or that cyclin E is released from the NM as a consequence of one of the events that lead to transformation. In fact, failure to recruit cyclin E (and by implication, initiation) to the NM in undifferentiated and cancer cells may be one of the factors that promote plasticity in response to extrinsic or intrinsic signals.

Overall, the work outlined here suggests that all three phases of DNA replication can occur in association with the NM. However, this may not be true for all cell types, making choice of experimental system crucial when planning further work. For various practical reasons, much of the analysis of DNA replication is undertaken with *Xenopus* eggs, or cancer cell lines such as HeLa, sometimes leading to the conclusion that NM immobilization may not be important for DNA replication. We would argue that the reported differences in nuclear organization in terms of proteins and loop attachments between embryonic systems and cancer cell lines on the one hand, and noncancer, differentiated cells on the other, offers a clear path forward.

## Models

Here we consider models that attempt to explain how DNA replication may be organized in relation to the NM. Template DNA and DNA replication enzymes must move relative to each other during synthesis. It is still common to see depictions that show the DNA replication machinery as an entity that moves along the DNA strand. However, consideration of the evidence (below) has lead to the suggestion that the DNA replication machinery is static and instead the DNA moves through these fixed sites (Pardoll *et al*. 1980).

### Replication factories

Replication foci or ‘factories’ can be visualized by immuno-detection of replication enzymes, or by following incorporation of nucleotide analogues into nascent DNA. They are believed to be macromole-cular assemblies populated by the enzymes that replicate DNA, forming several hundred efficient factories representing clusters of origins and that are activated at the same time. The number of origins is thought to be highly heterogeneous but to be an average of 5–6 per factory (Berezney *et al*. 2000; Frouin *et al*. 2003; and references therein). There is some reason to think that clustering of origins may offer an energy saving, as activation in near space may enable more efficient processing due to locally higher concentrations of factors (Frouin *et al*. 2003) (Fig. 2D). However, not all origins are activated at the same time in S-phase. Some areas of the genome are replicated early in S-phase while others are replicated later. Replication foci from all temporal stages of S-phase have been shown to be NM associated (Nakayasu & Berezney 1989) complete with nascent DNA which gradually moves out from each focus (Hozak *et al*. 1993, 1994), consistent with the concept of emanating loops. Cohesin is thought to help hold loops together because it is present at origins, interacts with the pre-RC, and its absence slows S-phase (Guillou *et al*. 2010). These data indicate that the replication machinery is located at NM attachment points at the base of chromatin loops.

### Organization of chromatin loops

Chromatin loops were first thought to be purely structural, giving way to more complex models with functionality at the base of loops. Depending on which direction researchers have approached this problem, models tend to appear in one of two forms, either a flower shaped factory of loops with little consideration of the NM (Frouin *et al*. 2003), or attachment to a NM at loop bases without representation of how these may come together into a factory (Cook 1999; Ottaviani *et al*. 2008) (Fig. 2B,D).

We, and others (Cook 1999; Ottaviani *et al*. 2008; Rivera-Mulia *et al*. 2011), hypothesize that recruitment of origins to the NM is part of the process of initiation (Fig. 2C). Recruitment appears to occur after pre-RC formation, as much of this is laid down at telophase in the mitotic cell cycle, yet origins appear to be recruited later in G1-phase (Djeliova *et al*. 2001b). When incorporating origin recruitment into models, several show an origin located distally on a loop, being recruited to the NM in late G1/S-phase. As DNA is spooled through replication factories, newly synthesized DNA is extruded as two new loops, with their origins returning to their original location (Cook 1999; Ottaviani *et al*. 2008). This implies a transient shortening of loops as origin attachments are made and a further transient shortening as DNA is reeled through replication machinery following origin activation. To understand the impact this would have on average loop size, and we must consider whether origins are all recruited at the same time because if this is not synchronous the effect on loop size could be minimal. Djeliova *et al*. (2001a,b) report that both an early and a late origin were recruited to the NM during late G1 (described in more detail above), suggesting that recruitment to the NM is synchronous rather than temporally distributed as for replication factory activation. However, analysis of chromatin loop size by MFHR has revealed no global loop remodeling between different phases of the cell cycle (Jackson *et al*. 1990).

To reconcile the model with these observations, we considered the consequences of recruitment of origins that are more proximal to existing attachment points, as proposed elsewhere (Rivera-Mulia *et al*. 2011). Recruitment of origins at the ‘top’ of loops would cause a reduction in loop size by up to 50%, but this would be the maximum observed. Recruitment of origins closer to the existing S/MAR would have less of an effect on loop size. For example, recruitment of origins a quarter of the way ‘up’ a loop would initially cause only a maximum of 12.5% drop in loop size (Fig. 2C), and the closer the origin is to an existing S/MAR the less the effect would be. Recruitment of origins close to existing S/MARs may preferentially occur, in which case a small decrease in global loop size may be difficult to observe and may explain why no decrease is reported for late G1-phase.

Similarly, no decrease in loop size is reported for S-phase despite a theoretical decrease, as the DNA is reeled in towards the replication machinery at the NM. This is perhaps not surprising given that origins are not all activated at the same time but as part of an organized temporal programe. Taken together, these arguments go some way to explaining little global impact on average chromatin loop size in either late G1 or S-phase.

### Similarities to transcription

Briefly, we highlight the data suggesting a role of the NM in transcription, as conclusions from this similar mechanism can be informative. There is a large array of published work implicating the NM as the site for transcription (summarised in Razin *et al*. 2011). Nuclear skeleton preparations showed nascent RNA, active RNA polymerase, and active genes in the resistant fraction, with similar results from NM preparations (Jackson & Cook 1985; and references therein). Consistent with this, several reports suggest that attachment points are enriched at transcribed genes (Jackson *et al*. 1996; Craig *et al*. 1997), with loops of ∼4 kb, in contrast to ∼200 kb loops of inactive chromatin (Bode *et al*. 2003), suggesting the attachments have functional significance. A proteomics screen of NM proteins returned many transcription factors (Albrethsen *et al*. 2009), which have been suggested to be MARBPs with binding sites potentially increasing the probability of a S/MAR. FISH to various genes showed transcriptionally active genes exhibited a spot at the NM (increased attachment) whereas transcriptionally inactive genes were extended on loops (Gerdes *et al*. 1994). However, this separation was less clear during S-phase when it was suggested that the attachment pattern was then a combination of effects of transcriptional status and transient NM attachment during replication with both gain and loss of attachments during this period (Gerdes *et al*. 1994). Models proposed for transcription, like replication, involve loops forming factories (Cook 1999; Sutherland & Bickmore 2009), implying a degree of commonality in mechanisms, but also a potential conflict of organization.

## Nuclear changes in cancer and disease

As a common feature of cancer cells, changes in nuclear architecture and morphology have long been the basis for cancer diagnoses (reviewed in Zink *et al*. 2004). However, without a complete understanding of nuclear architecture in normal cells it is difficult to consider whether such changes are cause or consequence of the dysregulation of cancer cells. In addition to genetic and epigenetic changes, there are many other recorded changes in the organization of genes, subnuclear domains, nonlinear DNA associations, and regulatory and macromolecular complexes in cancer cells (reviewed extensively elsewhere, for example, in Zaidi *et al*. 2007). This is also true for several other diseases (reviewed in Bode *et al*. 2000; Linnemann & Krawetz 2009a), including the thalassaemias where deletions often correspond with S/MAR sites suggesting that a change in chromatin loop attachment is a contributory factor (Bode *et al*. 2000). Changes in NM composition have been described for cancer cells compared to noncancer cells, with some exploited as the basis of diagnostic tests (Zink *et al*. 2004; He *et al*. 2008; Albrethsen *et al*. 2009; Lever & Sheer 2010; Munkley *et al*. 2011; and references therein). For example, a splice variant of the NM protein CIZ1 is associated with and can be detected in the blood of individuals with early stage disease (Higgins *et al*. 2012). Splice variants or altered expression of CIZ1 have also been reported to be associated with other cancers, including medulloblastoma, breast cancer, and Ewing's sarcoma (Warder & Keherly 2003; den Hollander & Kumar 2006; Rahman *et al*. 2007) and with other disorders including Alzheimer disease, rheumatoid arthritis, and cervical dystonia (Judex *et al*. 2003; Dahmcke *et al*. 2008; Xiao *et al*. 2012), many of which report alteration in subnuclear localization. The MARBP SATB1 is linked to aggressive breast cancers, (reviewed in Lever & Sheer 2010) while SAF-B is inversely correlated with the proliferative rate of tumors (reviewed in Lever & Sheer 2010). A number of NM proteins (for example, RUNX, CIZ1 and cyclin E) have been reported to have an altered localization in cancer cells, sometimes due to failure to be recruited to the NM (Zaidi *et al*. 2007; Munkley *et al*. 2011; Higgins *et al*. 2012).

We suggest that some of the dysregulation of cancer cells may be a direct result of events such as these, with DNA replication and transcription no longer spatially constrained by attachment to the NM. NM composition and loop attachments have been observed to change during differentiation and development (Getzenberg 1994; Munkley *et al*. 2011; Varma & Mishra 2011), leading to the idea that this may serve to ‘fix’ the developmental programe (Vassetzky *et al*. 2000; Munkley *et al*. 2011). We suggest this fixing of cell type specific characteristics such as transcriptional programe and replication timing is defective in cancer cells, resulting in a partial reversal to a less specified, embryonic-like organization of chromatin.

## Conclusions

The replication of DNA is fundamental to all cells, but is often deregulated in cancer cells with dramatic consequences. Data are amassing that suggest all stages of DNA replication are located at the NM at least in some cell types, and it is becoming clear that immobilization of DNA machinery and the resultant mechanisms of replication are likely to be an important method of regulating DNA synthesis. DNA replication should therefore not be considered alone, but in the context of nuclear architecture, with many other processes occurring at the same time and in the same space, in connection with a nuclear substructure. The NM can appear mysterious and elusive in detail, which we suggest may reflect the model system of choice.

The available published work suggests that in differentiated, noncancer cells, attachment of DNA and proteins involved in DNA replication to a NM occurs at specific times in the preparation for and during DNA replication. This is likely to constrain the process in time and space and possibly help to establish a heritable pattern of DNA replication. Attachment to a spatially constrained structure would also promote efficient use of required factors, with locally high concentrations at specific sites rather than diffusely spread throughout the nucleus. However, using the behavior of cyclin E as an indicator, we suggest that in undifferentiated or cancer cells initiation of DNA replication is less fixed in space. We suggest that failure to constrain initiation may allow greater flexibility and plasticity in these cell types, and that such flexibility may be lost with the imposition of spatial constraint as cells differentiate. This idea echoes the change in origin usage recorded as cells differentiate, with random and more frequent origin usage in early embryonic cell cycles, giving way to fewer and more specified origin usage in later cell cycles. Many other fundamental processes also appear to occur at the NM, such as transcription, DNA repair and epigenetic remodeling. Therefore, it is likely that these may also be subject to a similar level of regulation as DNA replication and their disregulation in cancer may similarly be in part due to compromised attachment to the NM.

As well as these potential differences between cell types we should also bear in mind that there may well be different mechanisms occurring within one cell. While there appears a universal process of DNA synthesis, it is likely that there will be differences in the initiation process. For example, there may be slightly different mechanisms used to select and initiate origins that govern replication through euchromatin compared to heterochromatin.

In conclusion we suggest that the relationship between the NM and DNA replication is important and complex, providing organization in many forms as well as anchoring key processes. However, this relationship cannot at the moment be generalized with differences occurring between cell types, and we hypothesize different types of chromatin within the same cell.
